# Exploring patterns in psychiatric outpatients’ preferences for involvement in decision-making: a latent class analysis approach

**DOI:** 10.1186/s12888-021-03137-x

**Published:** 2021-03-06

**Authors:** Ingunn Mundal, Mariela Loreto Lara-Cabrera, Moisés Betancort, Carlos De las Cuevas

**Affiliations:** 1 Faculty of Health and Social Sciences, Molde University College, Industriveien 18, Høgskolesenteret, 6517 Kristiansund, Norway; 2 Department of Mental Health, Faculty of Medicine and Health sciences, Norwegian University of Science and Technology (NTNU), Trondheim, Norway; 3 Kristiansund Community Mental Health Centre, Division of Psychiatry, Møre and Romsdal Hospital Trust, Kristiansund, Norway; 4 Tiller Community Mental Health Centre, Division of Psychiatry, St. Olav’s University Hospital, Trondheim, Norway; 5 Department of Research and Development, Division of Mental Health, St Olav’s University Hospital, Trondheim, Norway; 6 Department of Clinical Psychology, Psychobiology and Methodology, Universidad de La Laguna, San Cristóbal de La Laguna, Canary Islands Spain; 7 Department of Internal Medicine, Dermatology and Psychiatry, Universidad de La Laguna, San Cristóbal de La Laguna, Spain; 8 Instituto Universitario de Neurociencia (IUNE), Universidad de La Laguna, San Cristóbal de La Laguna, Spain

**Keywords:** Community mental health services, Latent class analysis, Mental disorders, Preferences, Private mental health service, Psychiatry, Shared decision-making

## Abstract

**Background:**

Shared decision-making (SDM), a collaborative approach that includes and respects patients’ preferences for involvement in decision-making about their treatment, is increasingly advocated. However, in the practice of clinical psychiatry, implementing SDM seems difficult to accomplish. Although the number of studies related to psychiatric patients’ preferences for involvement is increasing, studies have largely focused on understanding patients in public mental healthcare settings. Thus, investigating patient preferences for involvement in both public and private settings is of particular importance in psychiatric research. The objectives of this study were to identify different latent class typologies of patient preferences for involvement in the decision-making process, and to investigate how patient characteristics predict these typologies in mental healthcare settings.

**Methods:**

We conducted latent class analysis (LCA) to identify groups of psychiatric outpatients with similar preferences for involvement in decision-making to estimate the probability that each patient belonged to a certain class based on sociodemographic, clinical and health belief variables.

**Results:**

The LCA included 224 consecutive psychiatric outpatients’ preferences for involvement in treatment decisions in public and private psychiatric settings. The LCA identified three distinct preference typologies, two collaborative and one passive, accounting for 78% of the variance. Class 1 (26%) included collaborative men aged 34–44 years with an average level of education who were treated by public services for a depressive disorder, had high psychological reactance, believed they controlled their disease and had a pharmacophobic attitude. Class 2 (29%) included collaborative women younger than 33 years with an average level of education, who were treated by public services for an anxiety disorder, had low psychological reactance or health control belief and had an unconcerned attitude toward medication. Class 3 (45%) included passive women older than 55 years with lower education levels who had a depressive disorder, had low psychological reactance, attributed the control of their disease to their psychiatrists and had a pharmacophilic attitude.

**Conclusions:**

Our findings highlight how psychiatric patients vary in pattern of preferences for treatment involvement regarding demographic variables and health status, providing insight into understanding the pattern of preferences and comprising a significant advance in mental healthcare research.

## Background

Shared decision-making (SDM) is a collaborative, patient-centred approach in which clinicians and patients share the best available evidence when faced with the task of making decisions and in which patients are supported in considering options to achieve informed preferences [1]. Shared decision-making is an emerging area of interest in psychiatry. The scientific literature of the last decade has produced a remarkable proliferation of publications presenting rationales for SDM in mental healthcare, but the evidence for SDM’s impact on clinical and patient-reported outcomes and care experiences is currently limited [2–4]. The involvement of psychiatric patients in SDM complies with the ethical principle of autonomy (the legal requirement of informed consent) and is associated with greater patient satisfaction; nonetheless, changes in practice are still governed by factors such as cost, profit margin, quality and efficiency [5]. Moreover, patients’ involvement in treatment might be considered disruptive since it demands a considerable shift in the power and control of interactions between clinicians and patients through collaborative decision-making and implies a change in the way clinical psychiatry is practiced [6]. Involvement may also depend on potential barriers to SDM in psychiatric care, such as patients’ decision- making capacity or therapeutic style and setting [7].

The practice of psychiatry is characterised by many clinical situations in which there are multiple reasonable possibilities for intervention and no evidence of the supremacy of one approach over another. There is more than one appropriate response for each mental health problem, and decisions are considered ‘preference-sensitive’ because patients may have more than a ‘single’ best choice [8, 9]. For example, the Spanish healthcare system is based on the principles of universality, free access, equity and fairness of financing, and it is mainly funded by taxes [10]. Patients who require mental health treatment typically receive care through family medicine doctors, while those with serious or ongoing illnesses are referred for specialist treatment provided at community mental health centres. They may also access private treatment not covered by state health insurance, and patients need private coverage if they want to avoid paying the full costs of mental health services. Many psychiatrists working in the public sector also provide private consultations. Environmental, contextual and mental health expectations in psychiatric patients as well as waiting times and frequency of follow-up consultations may differ between the public and private sectors.

Patients’ perceptions of care quality are essential indicators reflecting patients’ perceptions of standards in healthcare, and these perceptions also clarify how patients define quality [11]. Although the research literature considers SDM to be a best practice in mental healthcare [12] that patients value [2], more research is needed regarding psychiatric patients’ preferences for and experiences of SDM. Thus far, little is known about the psychological factors conditioning patients’ preferences regarding SDM. Understanding psychiatric patients’ preferences for involvement in decision-making for treatment is relevant to improving their self-determination and empowerment and to further developing psychiatric services [5]. Moreover, this insight would support patients in making informed healthcare decisions that are consistent with their needs, values and preferences and that consider the potential benefit and risk trade-offs of different options [8]. Despite the increased attention to this topic, available empirical evidence base regarding involvement in treatment-related decisions is inconclusive [13]. Although SDM is recommended in mental health systems, available evidence suggests that SDM in mental healthcare is particularly challenging and is not yet widely implemented [13]. Clinicians may find SDM difficult to achieve, and most healthcare systems do not view this approach as the standard of care. Thus, patient preferences should be a particularly important consideration in the healthcare decision-making process.

In terms of psychiatric patients’ beliefs about and perceived control within the context of health, their ability to positively influence their own health is among the more reliable determinants of health behaviour and health outcomes [14]. Such determinants are in accordance with the health belief model used to explain and predict health-related behaviours [15, 16]. They may also be related to attitudes toward psychiatric medications [14], health locus of control (the beliefs patients have about who or what is the agent that determines the state of their health) [17] and psychological reactance (the emotional reaction against rules or regulations that threaten or suppress certain freedoms of behaviour) [18]. These psychological variables may be relevant covariates in understanding psychiatric patients’ preferences for involvement in decision-making–covariates that have not been sufficiently elaborated and thus have not been investigated [19].

Therefore, the objectives of this study to are to identify different latent class typologies of patient preferences for involvement in decision-making and to investigate how patient characteristics predict which of these classes they belong to in both mental healthcare settings.

## Methods

### Sample

From November 2019 to January 2020, we invited 300 consecutive psychiatric outpatients treated in two outpatient healthcare departments – one public, belonging to the Canary Islands Health Service, and the other private – to participate anonymously in the study. Patients at both sites were offered the same types of mental health treatment options on an outpatient basis, which included individual psychotherapy, group therapy, medication and medical supervision*.* However, the private and public psychiatric practice differ in that private practice is characterised by shorter waiting times, greater continuity and lasting therapies, the possibility to choose the therapy and therapist and more privacy. While public healthcare is free of charge for the patient, the patient must pay for the cost of treatment and care in a private practice.

The second author fully explained the study to each participant in the waiting room before the participant’s consultation. Patients were eligible for inclusion if they were 18 years or older, receiving psychiatric treatment, were able to read and write in Spanish and were able to provide written informed consent. Patients younger than 18, illiterate patients and those seriously cognitively impaired were excluded. All participants signed an informed consent form. Each participant then filled out a brief sociodemographic survey and the remaining questionnaires. Because of the anonymous design of this study, we did not gather information on those who chose not to participate.

### Measures

#### Patients’ preferences regarding involvement in decision-making

The psychiatric outpatients’ preferences related to involvement in decision-making on their treatment were measured using the Spanish-language validated version of the Control Preferences Scale (CPS) [20, 21]. This is the most frequently used instrument for assessing patient preferences related to being involved in decisions about their health [22], and it has shown a moderate level of internal consistency (Cronbach’s α = 0.72) [20]. In this study, we used the card-sorting version of the CPS, which is a self-administered version of the scale consisting of five illustrated vignettes representing different roles in decision-making, with a short descriptive statement under each illustration [23]. The patient reports the two most preferred roles, resulting in six possible scores: active–active, active–collaborative, collaborative–active, collaborative–passive, passive–collaborative and passive–passive. These scores are grouped as active (active–active or active–collaborative), collaborative (collaborative–active or collaborative–passive) or passive (passive–collaborative or passive–passive).

#### Patients’ health beliefs questionnaire on psychiatric treatment (PHBQ)

Psychiatric patients’ health beliefs were assessed using the Patient’s Health Belief Questionnaire on Psychiatric Treatment (PHBQ) [14], a 17-item self-reported health beliefs scale that integrates three concepts of attitudes toward psychiatric medication, locus of health control and psychological reactance and which predicts patient adherence to prescribed medications. The questionnaire includes five meaningful subscales: a) internal health locus of control, which is the belief that one’s own behaviours affect one’s mental health status; b) doctor health locus of control, which is the belief that doctors determine the outcomes of a patient’s mental health; c) psychological reactance, which is the patient’s motivation to regain a freedom after it has been lost or threatened, leading patients to resist the influence of mental health professionals; d) positive aspects of medication, which describes positive attitudes toward psychiatric medications; and e) negative aspects of medication, which describes negative attitudes toward prescribed psychotropic. The internal health locus, doctor health locus, psychological reactance and negative aspects of medication subscales each include three items, whereas the positive aspects of medication subscale comprises five items, thus totalling 17 items on the questionnaire.

Patients were asked to use a 6-point Likert scale from 1 (totally disagree), to 6 (totally agree) to rate the extent to which they agree or disagree with each statement. Higher scores on each subscale indicate a stronger belief. Participants in the study were classified according to their scores on each subscale and categorised based on the 33rd and 66th percentiles into three categories: high, medium and low.

A recent study of the preliminary potential of the PBHQ found Cronbach’s α coefficients were 0.67 for the internal health locus of control subscale; 0.65 for the doctor health locus of control subscale, 0.67 for the psychological reactance subscale, 0.70 for the positive aspects of medications subscale and 0.68 the negative aspects of medications subscale [14].

### Data analysis

Statistical analyses were performed using the software program IBM SPSS Statistics version 25 [24]. Fulfilment of assumptions of normality and homogeneity were tested prior to the application of analyses. The Mann–Whitney U test was performed to analyse differences between two independent groups when the dependent variable was either ordinal or continuous but not normally distributed. Differences between groups in categorical variables were analysed using a chi-square test when the cell sizes were large and using Fisher’s exact test for small samples. Multivariate analyses were carried out for those variables that in the univariate analyses showed a significant relationship to both the preferences for and experiences of the decision-making process. Logistic regression analyses were performed to identify factors associated with preferences for involvement in decision-making and experiences. For univariate analyses and correlations, Pearson’s correlation coefficients and analysis of variance were used.

To explore preference heterogeneity, we conducted latent class analyses (LCAs) using the poLCA package in R. Latent class analysis is an emerging technique used in stated-preference studies to segment people by preferences instead of observed characteristics, and is based on peoples’ scoring patterns across variables rather than being driven by associations with an outcome [25, 26]. Segmentation is an alternative to stratification, and respondents are classified into groups or clusters based on the patterns of choices or preferences [27]. An LCA was carried out to identify latent classes of psychiatric outpatients with distinct patterns of involvement preferences in decision-making with similar preferences of involvement in decision-making, estimating the probability that each patient belongs to a given class based on sociodemographic, clinical and health belief variables. This LCA was explicitly designed to identify different ‘classes’ of patients and to examine the unique characteristics of each class, with members within a class being relatively similar and those across classes being relatively dissimilar regarding preferences for involvement in decision-making. We established three latent classes a priori according to levels of the theoretical output variable ‘preferred involvement’ in decision-making. Five unconditional models with from one to five latent classes were tested. The search algorithm produced 20 models for each class and up to 3000 iterations to obtain maximum likelihood. The models were evaluated based on the adjustment criteria of the minor Bayesian information criterion (BIC), the adjusted BIC, the adjusted Aikake information criteria (AIC), and the Lo-Mendell-Rubin adjusted likelihood ratio tests to assess adequate separation of classes. Entropy indicated the accuracy of classification with a larger value.

The nature of LCA is to search for profiles (latent classes) that are related to a set of multivariate variables of an exclusive (categorical), discrete nature. In our LCA, we included all the variables measured in patients, including the site variable, in order to elaborate possible decision-making profiles from the combinations of responses to these variables.

## Results

A response rate of 74.6% resulted in a sample of 224 psychiatric outpatients (159 from public psychiatry facilities and 72 from private practices) with no missing items, which demonstrated the acceptability of the self-reported data.

Table 1 provides a descriptive analysis comparing sociodemographic and clinical variables from psychiatric outpatients from public and private settings as well as their preferred roles in decision-making and their health beliefs. Chi-squared tests were used to compare prevalence, and a nonparametric Mann–Whitney U test compared continuous variables. Most patients in the study were female (62.1%), and the mean age was 44.4 ± 15.1 years. As for participants’ education level, 24.6% had completed primary education, 46% had completed secondary education, and 29.5% had a university degree. Diagnoses were available for 90% of patients, with depressive disorder the most prevalent diagnosis (39.8%), followed by anxiety disorders (39.3%), schizophrenia (11.9%) and bipolar disorders (5%). According to the CPS results, almost one half of patients (107, 48.9%) preferred a collaborative role in decision-making wherein the doctor and patient share responsibility for deciding which treatment is best, while 92 (42%) preferred a passive role and only 20 (9.1%) an active role.

**Table 1:** Sociodemographic and clinical profiles of the samples studied (*n* = 224; 159 public psychiatry patients and 72 private psychiatry patients)

	Global	Public	Private	*P* value
Women, n (%)	139 (62.1)	94 (61.8)	45 (62.5)	.523^a^
Mean age (SD)	44,4 (15.1)	41,2 (13.9)	51,3 (15.1)	**<.001**^**b**^
Education level, n (%)				
*Primary studies*	55 (24.6)	37 (24.3)	18 (25.0)	**.004**^**a**^
*Secondary studies*	103 (46.0)	80 (52.6)	23 (31.9)	
*University degree*	66 (29.5)	35 (23.0)	31 (43.1)	
Time as psychiatric patient (moths), mean (SD)	77,9 (106)	73.3 (104)	89.8 (110)	.340^b^
Number of psychiatric drugs used, mean (SD)	1.7 (1.3)	1.88(1.4)	1.85(.9)	.855^b^
Diagnosis, n (%)				
*Schizophrenia*	24 (11.9)	18 (14.0)	6 (8.3)	.546^a^
*Bipolar disorder*	10 (5.0)	6 (4.7)	4 (5.6)	
*Depressive disorder*	80 (39.8)	53 (41.1)	27 (37.5)	
*Anxiety disorder*	78 (39.3)	46 (35.7)	33 (45.8)	
*Personality disorder*	8 (4.0)	6 (4.7)	2 (2.8)	
Preferences of Involvement in SDM, according CPS, n (%)				
*Active*	**20 (9.1)**	**18 (12.1)**	**2 (2.9)**	**<.001**^**a**^
*Active-Active*	5 (2.3)	4 (2.7)	1 (1.4)	
*Active-Collaborative*	15 (6.8)	14 (9.4)	1 (1.4)	
*Collaborative*	**107 (48.9)**	**81 (54.4)**	**26 (37.1)**	
*Collaborative-Active*	21 (9.6)	13 (8.7)	8 (11.4)	
*Collaborative-Passive*	86 (39.3)	68 (45.6)	18 (25.7)	
*Passive*	**92 (42.0)**	**50 (33.6)**	**42 (60.0)**	
*Passive-Collaborative*	64 (29.2)	38 (25.5)	26 (37.1)	
*Passive-Passive*	28 (12.8)	12 (8.1)	16 (22.9)	
Health Beliefs Questionnaire Dimensions, mean (SD)				
*Internal health locus of control*	13.0 (4.0)	12.7 (4.0)	13.6 (3.9)	.119^b^
*Doctors health locus of control*	14.6 (3.7)	14.1 (3.8)	15.6 (3.1)	**.003**^**b**^
*Psychological reactance*	10.3 (3.7)	10.5 (3.8)	9.9 (3.6)	.280^b^
*Positive aspects of medications*	20.2 (6.3)	19.0 (6.2)	22.8 (5.7)	**<.001**^**b**^
*Negative aspects of medications*	9.5 (4.3)	9.4 (4.4)	9.6 (4.2)	.642^b^

^a^ Chi-square

^b^ Mann–Whitney U

*Abbreviations*: *n* number of patients, *SD* standard deviation, *SDM* Shared Decision-Making, *CPS* Control Preferences Scale, *Sig* significance

Private practice psychiatric outpatients were significantly older than patients in public practice (public = 41.2 ± 13.9 years, private = 51.3 ± 15.1, F = 24.378, *p* < .001). Patients in private practice reported higher educational levels (X^2^ = 11.208, *p* = .004), preferred a significantly more passive role in decision-making regarding their treatment (X^2^ = 20.291, *p* = .001) and registered higher scores on the doctor health locus of control subscale (public = 14.1 ± 3.8, private = 15.6 ± 3.1, F = 9.063, *p* = .003), meaning higher attribution of their mental health as dependent upon the actions of their psychiatrist. Moreover, these patients also had higher scores on the positive aspects of psychiatric medication (public = 19.0 ± 6.2, private = 22.8 ± 5.7, F = 18.410, *p* < .001), indicating a more positive attitude toward medication among patients in private psychiatric practices than among those in public practices.

A group of analyses were carried out to determine if preference groups or health beliefs groups differed in terms of sociodemographic or clinical variables (data shown in Tables 2, 3 and 4). In a univariate analysis of patients’ preferences of involvement in decision-making, age showed statistical significance (active = 36.5 ± 17.8, collaborative = 42.4 ± 14.6, passive = 48.6 ± 14.3, F = 7.521, *p* = .001), with older patients preferring more passive roles. The chi-squared analysis indicated level of education was statistically significant (X^2^ = 22.798, *p* < .001) in patients’ preferences of involvement. Patients with lower education levels mostly preferred a passive role (65.4%), whereas there was a general tendency toward a more collaborative preference as education level increased. A univariate analysis of health belief dimensions found that only internal health locus of control varied significantly according to patients’ gender (male 13.9 ± 3.7 vs. female 12.4 ± 4.0, F = 8.001, *p* = .005). Age was significantly correlated with doctor health locus of control (Pearson = .246, *p* < .000), psychological reactance (Pearson = −.182, p = .005) and positive aspects of medication (Pearson = .328, p < .000). Patients’ diagnoses showed significant differences in doctor health locus of control (schizophrenia 15.6 ± 2.4, bipolar disorder 15.6 ± 3.1, depressive disorder 15.2 ± 3.5, anxiety disorder 13.8 ± 4.2, personality disorder, 12.1 ± 4.5, F = 3.020, *p* = .019) and positive aspects of medication (schizophrenia 23.0 ± 6.7, bipolar disorder 25.5 ± 2.6, depressive disorder 21.0 ± 5.7, anxiety disorder 18.8 ± 6.4, personality disorder, 18.4 ± 4.2, F = 4.754, *p* = .001). Finally, only positive aspects of medication registered significant differences in relation to the preferred role of involvement (active 17.2 ± 6.4, collaborative 19.6 ± 6.1, passive 21.4 ± 6.3, F = 4.599, p = .01), with passive patients registering the highest scores in this dimension.

**Table 2:** Univariate association between the Patients’ preference of involvement, age and education

Variables	Preference group	Mean	SD	***F***	***P***-value
Age (years)	Active	36.5	±17.8	F = 7.521	*p* = 0.001
	Collaborative	42.4	±14.6		
	Passive	48.6	±14.4		
Education		X^2^ = 22.798,			p < .001
Positive aspects of medication				F = 4.599	*p* = 0.01
	Active	17.2	± 6.4		
	Collaborative	19.6	± 6.1		
	Passive	21.4	± 6.3		

*Note*: *SD* Standard Deviation

**Table 3:** Univariate association between the PHBQ and Gender and Age

Item	Mean	SD	ANOVA results/correlations	
^G^Internal health locus of control			F = 8.001	0.005
Male	13.9	± 3.7		
Female	12.4	± 4.0		
^A^Doctor health locus of control			Pearson = .246, *p* < .001	
^A^Psychological reactance			Pearson = −.182, *p* = 0.005	
^A^Positive aspects of medication			Pearson = .328, p < .001	

Note: *SD* Standard Deviation. ^G^Gender, ^A^Age; PHBQ: Patient’s Health Belief Questionnaire on Psychiatric Treatment

**Table 4:** Univariate association between the PHBQ and diagnoses

	Mean	SD	***F***	***P***-value
**Doctor health locus of control**			F = 3.020	*p* = 0.019
Schizophrenia	15.6	± 2.4		
Bipolar disorder	15.6	± 3.1		
Depressive disorder	15.2	± 3.5		
Anxiety disorder	13.8	± 4.2		
Personality disorder	12.1	± 4.5		
**Positive aspects of medication**			F = 4.754	p = 0.001
Schizophrenia	23.0	± 6.7		
Bipolar disorder	25.5	± 2.6		
Depressive disorder	21.7	± 5.7		
Anxiety disorder	18.8	± 6.4		
Personality disorder	18.4	± 4.2		

*Note*: *SD* Standard Deviation, *PHBQ* Patient’s Health Belief Questionnaire on Psychiatric Treatment

The values of the fit indicators for comparing models in the LCA are reported in Table 5. The smallest of the three aforementioned fit indicators and the higher entropy supported the superiority of a three-class model over the alternatives. This best fit model included 192 participants, 74 estimated parameters and 118 degrees of freedom and obtained reasonable adjustment indices and an adequate entropy level close to 1, which explained 78% of the frequency variability in the categories of the variables of interest.

**Table 5:** Values of the fit indicators for model comparisons in latent class analysis

Model	log- likelihood	Residual degree of freedom (df)	BIC	aBIC	aAIC	Likelihood-ratio	Entropy
Model 1		168	4503.24	4427.22	4527.24	2360.96	–
Model 2		143	4506.82	4351.60	4555.82	2233.10	0.710
Model 3		118	4553.51	4319.10	4627.51	2148.35	0.780
Model 4	−2.051.348	93	4623.19	4309.59	4722.19	2.086.59	0.843
Model 5	−2.024.382	68	4700.69	4307.90	4824.69	2.032.66	0.854

*BIC* Bayesian information criterion, *aBIC* Adjusted Bayesian information criterion, *aAIC* Adjusted Aikakes’ information criteria; Likelihood ratio: Lo-Mendell-Rubin adjusted likelihood ratio tests

Results of the LCA indicated that three latent classes provided the best fit for the data. Table 6 shows the conditional probabilities of each category for the variables of interest in the three classes. The LCA generated three different psychiatric patient profiles of preferences for involvement in decision-making – two collaborative and one passive – based on the conditional probabilities of co-occurrence of the categories in the variables of interest. The analysis initially showed that the highest probability (45%) of class membership in our sample was for class 3, with a passive preference of involvement. This profile included women older than 55 (56%) with a primary level education (41%) and depressive disorders (38%). It also described low psychological reactance (41%) and a greater external health locus of control characterised by high doctor health locus of control (48%) and an average internal health locus of control (37%). It also reported greater trust in psychotropics, as defined by a high assessment of positive aspects of psychiatric medication (59%) and an average consideration of their negative aspects (37%), indicating a pharmacophilic tendency.

**Table 6:** Conditional category response probabilities, by variable, for each class

Variable	Category	Type of Classes		
		Class 1 (*N* = 50)	Class 2 (*N* = 57)	Class 3 (*N* = 87)
*Conditional Probability*		***.26***	***.29***	***.45***
Health Care System	Public	.**69**	.**85**	**.48**
	Private	.30	.14	.**51**
Gender	Male	.**55**	.23	.33
	Female	.44	.**76**	.**67**
Age	18–33 years	.32	.**43**	.02
	34–44 years	.**34**	.28	.16
	45–54 years	.27	.23	.23
	≥ 55 years	.05	.04	.**56**
Educational level	Primary	.0	.14	.**41**
	Secondary	.**66**	.**64**	.23
	University	.33	.21	.34
Diagnosis	Schizophrenia	.13	.0	.19
	Bipolar Disorder	0	0	.11
	Depressive Disorder	.**46**	.34	.**38**
	Anxiety Disorder	.36	.**55**	.30
	Personality Disorder	.03	.09	0
Internal Health Locus of Control	Low	.03	.**58**	.32
	Medium	.45	.20	.**37**
	High	.**50**	.21	.29
Doctor Health Locus of Control	Low	.0	.**79**	.22
	Medium	.**65**	.20	.29
	High	.34	0	.**48**
Psychological Reactance	Low	.10	.**45**	.**41**
	Medium	.30	.29	.38
	High	.**59**	.25	.19
Positive Aspects of Medication	Low	.22	.**68**	.15
	Medium	.**59**	.25	.25
	High	.18	.06	.**59**
Negative Aspects of Medication	Low	.22	.**47**	.33
	Medium	.21	.29	.**37**
	High	.**56**	.23	.28
Preference of Involvement	Active	.06	.09	.06
	Collaborative	.**66**	.**61**	.30
	Passive	.26	.28	.**62**

Note: Latent class and conditional probability for proportion of participants in class. Bold numbers are high conditional probabilities that characterize each class. **Class 1**: Collaborative men attending public services for depression. **Class 2**: Collaborative young women attending public services for anxiety. **Class 3**: Passive preference among older women with low education and depression

The second highest probability (29%) of class membership was for class 2. This profile described collaborative women younger than 33 (76%) who were cared for in a public setting for an anxiety disorder (55%) and had low psychological reactance (45%), low internal health locus of control (58%) and low doctor health locus of control (79%).

**Fig. 1:**
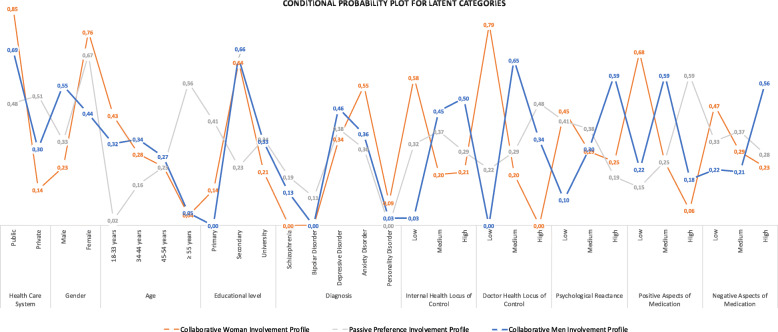
Conditional probability plot for the three latent classes for involvement. Collaborative women (red), Passive preference (grey), and Collaborative men (blue)

The last class, class 1, was identified with a 26% probability of class membership for class 1 and generated a profile of collaborative men (55%) aged 34 to 44 years (34%) who were cared for in a public setting for a depressive disorder (46%) and had high psychological reactance (59%), medium internal health locus of control (50%) and a high negative attitude towards medication (56%) that combined with a medium positive attitude towards medication (59%), representing a tendency for pharmacophobia. A conditional probability plot for the latent classes is shown in Fig. 1.

## Discussion

To our knowledge, this is the first study to employ advanced statistical techniques to specifically identify patterns or classes regarding psychiatric patients’ preferences for involvement in the decision-making process. We also investigated how patient characteristics are important in predicting their membership in one of these classes. To identify preference heterogeneity, we conducted LCAs. Three distinct preference classes were identified – two collaborative and one passive – accounting for 78% of the variance. Taken together, the results highlight the complexity of psychiatric patients’ preferences for involvement in decision-making related to their treatment. Accordingly, they provide relevant information about these preferences as well as give insight into how sociodemographic, clinical and health belief factors may affect patients’ preferences in both public and private mental healthcare settings.

### Treatment context and associations with SDM

We found several differences between the public-setting and private-setting patient samples. Patients from the private healthcare settings were older and preferred passive involvement roles in decision-making. These patients also held a more positive attitude towards medication. Our findings may rest on several circumstances and aspects that influence patients’ preference for decision-making, such as those related to care providers’ paternalistic views or decision style [28], the clinical experience, available resources [29], time constraints [30] and other related circumstances that may not be apparent to us. Other studies from public mental healthcare settings suggest that public psychiatry tends to rely more heavily on pharmacological treatment, provides shorter consultations, and has less continuity regarding the healthcare workers who treat a given patient [31, 32]. Additionally, pharmacological treatment is more frequently combined with psychotherapy in private settings [32]. Research on SDM in private mental healthcare settings is scarce, and more research is needed to better understand how patient preferences may differ between private and public healthcare settings.

A number of studies have examined the implementation of patient decision aids, aiming to empower patients to become more active and self-confident [33, 34] and conversation aids to promote patient-clinician interactions consistent with SDM [8]. However, there are important limitations in the evidence regarding SDM tools such as decision aids and whether these tools improve the situation of patients in a way that makes intellectual, emotional and practical sense to them [34]. To develop and implement approaches that are likely to improve SDM in mental health settings, further follow-up studies are warranted. We speculate that the differences in time and treatment approaches in mental health settings may explain some of the differences found in our study.

### Associations between preferences for involvement in decision-making, health belief dimensions and other variables of interest

We found psychiatric outpatients’ preferences for involvement in decision-making regarding their treatment to be significantly related to their age and level of education. Older patients preferred roles that were more passive – an observation in agreement with similar results reported by other authors [35]. Most patients with lower educational levels preferred a passive role, whereas there was a general tendency towards more collaboration as educational level increased. These results are in line with those of other authors who found that patients with higher levels of education tend to prefer a more active role in, for example, medical decisions, while those with fewer years of education may feel less confident about involvement in decision-making [35–37]. The findings can also be explained on the basis of potential barriers to SDM in psychiatric care. Hamann et al. [38] similarly found that providers of psychiatric services reported being more likely to use SDM for patients who were more adherent to treatment and had higher educational levels. Another study found that older healthcare providers used less SDM with patients in depression care [39]. However, we found no significant associations between gender, diagnosis or time in psychiatric treatment and involvement preferences.

Concerning health beliefs, only positive aspects of medications showed significant differences in relation to preferred level of involvement, with patients with a passive preference registering the highest scores in this dimension. SDM may be particularly relevant regarding preferences for sensitive decisions where there are several reasonable treatment options, and medication decisions and psychiatric medication management may fall in to this category as an important area of decision making [3, 40]. Consistent with the findings of De las Cuevas and Peñate (2016), most patients with emotional disorders express their preference for involvement in decision-making in a collaborative way when discussing available treatment options; however, they prefer that their psychiatrists make final decisions on their behalf [20]. This may be because patients in specialised public psychiatric care generally often experience more complex and chronic conditions, followed by uncertainty regarding treatment outcomes influenced by illness severity, their decision-making capacity, treatment availability and clinicians’ preference [41].

### Results of the latent class analysis

Latent class analysis is increasingly used to study preference heterogeneity in health and to support decision-making [26]. This technique allowed us to identify groups of psychiatric patients who shared common characteristics in such a way that patients within a group had a similar scoring pattern on the measured variables, while the difference in scoring patterns between the groups was as distinct as possible [25, 26]. Although the profiles generated by our analysis did not include an active preference profile, since our sample contained few patients that fit this profile, we believe our results underline an additional insight by accounting for preference heterogeneity. In contrast to descriptive approaches, the likelihood of misclassification in LCA can be quantified and estimated using goodness-of-fit tests and average posterior probabilities. We used AIC and BIC to determine the number of latent classes. However, LCA does not necessarily provide a firm answer for how many latent classes exist but rather acknowledges that other criteria exist, and alternative methods may have resulted in a different class structure [25, 26]. Accordingly, further examination of distinct patterns in patients’ preferences of involvement in decision-making may be better tailored in a larger-scale study.

### Clinical relevance

Traditionally, diagnosis is the source for decision-making in clinical practice, providing key information for clinical decisions that influence outcomes in serious acute illness [42]. Yet the central role of diagnosis is challenged by evidence that patient prognosis is influenced by more than disease diagnosis and diagnosis-driven treatment [43, 44]. Involvement in decision-making and patient preferences presents challenges in clinical practice and poses important implications for the management of healthcare. Like many other health conditions, psychiatric conditions are influenced by biological, psychological and social factors that interact to determine individuals’ prognoses and likely treatment responses [42]. Our study indicates differences between public and private psychiatric care, with older age, a higher level of education and a passive role in SDM associated with treatment in private care. Patients in private care also showed a more positive attitude towards medication. Psychiatric disorders often involve complex perceptual experiences at different stages of an illness, which may temporarily imply lack of insight, treatment adherence and decision-making capacity [14, 45, 46]. These patients mostly remain in public psychiatric care [32, 47]. We presume that our study addresses an important aspect of involvement in decision-making in that the patients’ preference for involvement may also reflect the therapist’s attitude to patient involvement. If the patient presents a passive preference for involvement, this may depend on the patient but also the therapist. Our study provides insights into understanding the pattern of the psychiatric preferences for involvement in treatment and is thus a significant advance in research in mental healthcare settings.

A significant preference heterogeneity may exist among patients based on different socioeconomic backgrounds, cultures, experiences, beliefs, personalities, clinical pictures of the disease or case histories [26, 48]. Our results highlight the complexity of psychiatric patients’ preferences of involvement in decision-making related to their treatment. The results provide relevant information about these preferences as well as about how sociodemographic, clinical and health belief factors may affect patients’ preferences in both public and private mental healthcare settings. This information will enable mental health professionals to empower psychiatric patients through interventions tailored to their preferences. Thus, our study meets the need to better understand how psychiatric patients perceive the decision-making process even though they may not wish to make the final decision [13].

Several limitations of this study must be considered. Firstly, the patient sample may be considered a convenience sample as only patients from public community mental health centres within the public Spanish National Healthcare System and private psychiatric clinics were recruited; this may limit generalisability of this research. Secondly, the patient samples were relatively small, which resulted in few cases that revealed distinct patterns in the LCA. Thirdly, the cross-sectional design required the results to be interpreted cautiously because it increased the difficulty of assessing whether the data reflected a trend or any kind of difference between the sample groups [49]. Finally, because of the anonymous design of this study, we did not collect information on those who chose not to participate. Such information could have provided valuable insight into factors useful to clinicians and policymakers developing interventions to improve involvement.

## Conclusion

In the present study, we explored the typology and potential predictors of psychiatric outpatients’ preferences for involvement in decision-making regarding their prescribed treatment in public and private mental health settings. Psychiatric outpatients preferred collaborative–passive roles in decision-making. The LCA demonstrated sociodemographic, clinical and health beliefs relevant to differences in the patients’ preferences for involvement in decision-making. Our study provides insights helpful to understanding the pattern of preferences for involvement in psychiatric treatment decisions and is thus a significant advance in research in mental healthcare settings. Interventions to empower psychiatric patients should be tailored according to patients’ preferences.

## Data Availability

The datasets used and/or analysed during the current study are available from the last author on reasonable request.

## Abbreviations

aAIC: Adjusted Aikakes’ information criteria
aBIC: Adjusted Bayesian information criterion
BIC: Bayesian information criterion
CPS: Control Preferences Scale
LCA: Latent Class Analysis
PHBQ: Patients’ Health Beliefs Questionnaire on Psychiatric Treatment
SD: Standard deviation
SDM: Shared decision-making
Sig: Significance
